# The little tissue that couldn’t – dispelling myths about the Hymen’s role in determining sexual history and assault

**DOI:** 10.1186/s12978-019-0731-8

**Published:** 2019-06-03

**Authors:** Ranit Mishori, Hope Ferdowsian, Karen Naimer, Muriel Volpellier, Thomas McHale

**Affiliations:** 1 Department of Family Medicine, Georgetown University School of Medicine, Washington, DC USA; 2 Department of Medicine, University of New Mexico School of Medicine, Albuquerque, NM USA; 3 Physicians for Human Rights, Program on Sexual Violence in Conflict Zones, Boston, MA USA; 4 Sexual Assault Referral Centre, The Havens Paddington, Kings College Hospital NHS Trust, London, UK

## Abstract

Conclusions about women’s and girls’ sexual history are made in some settings based on assumptions about the hymen, a small membranous tissue with no known biological function, which typically occupies a portion of the external vaginal opening in females. Clinicians, however, continue to refer to changes in the hymen to assess for a history of consensual or nonconsensual sexual intercourse. We reviewed published evidence to dispel commonly held myths about the hymen and its morphology, function, and use as evidence in cases of sexual violence.

An examination of the hymen is not an accurate or reliable test of a previous history of sexual activity, including sexual assault. Clinicians tasked with performing forensic sexual assault examinations should avoid descriptions such as “intact hymen” or “broken hymen” in all cases, and describe specific findings using international standards and terminology of morphological features.

We call on clinicians to consider the low predictive value of a hymen examination and to: 1) avoid relying solely on the status of the hymen in sexual assault examinations and reporting; 2) help raise awareness of this issue among their peers and counterparts in law enforcement and the judicial system; and 3) promote fact-based discussions about the limitations of hymenal examinations as part of clinical education for all specialties that address the sexual or reproductive health of women and girls.

**Electronic supplementary material:**

The online version of this article (10.1186/s12978-019-0731-8) contains supplementary material, which is available to authorized users.

## Plain English Summary

In some settings, clinicians who evaluate women and girls suspected of being victims of sexual assault, or suspected of having engaged in intercourse (with or without consent), rely on an examination of the hymen for their assessments. The hymen is a small membranous tissue outside of the vaginal canal that has no known biological function. We reviewed published studies about the hymen to help guide clinicians in evaluating whether or not a hymen examination would be a valuable practice.

We concluded that an examination of the hymen is not an accurate or reliable test of sexual activity, including sexual assault, except in very specific situations. Clinicians who perform forensic sexual assault examinations should avoid descriptions such as “intact hymen” or “broken hymen” in all cases, and describe specific clinical findings using specific medical terminology.

We recommend that clinicians take into consideration that a hymen examination does not generally offer a high degree of certainty about sexual activity, with or without consent. We call on clinicians to: 1) avoid relying solely on the status of the hymen in sexual assault examinations and reporting; 2) help raise awareness of this issue among their peers and counterparts in law enforcement and the judicial system; and 3) promote fact-based discussions about the limitations of hymenal examinations with their colleagues and health professional students from all specialties that address the sexual or reproductive health of women and girls.

## Background

In many patriarchal cultures, the sexual history of girls and women is used as a significant determinant of their societal, community, family, and individual status [1, 2]. Frequently, conclusions about sexual history are made based on assumptions about the hymen, a small membranous tissue with no known biological function [3, 4], which typically occupies a portion of the external vaginal opening in females.

The sociocultural significance of the hymen in certain communities as “proof” of the absence of sexual activity has even led to an intersection of culture, religion, politics, law, economics, and medicine in the form of “virginity testing,” which involves the use of a vaginal examination to evaluate whether or not a woman’s hymen is “intact,” in an attempt to ascertain whether a woman has had sexual intercourse [5, 6]. This is frequently done prior to marriage. Health care providers conduct and make conclusions about whether or not a woman or girl has had sexual intercourse based on these examinations [6, 7].

In some areas of the world, girls and women are subjected to an examination of the hymen to prove they have experienced sexual violence, as in the case of Yezidi women who were kidnapped, enslaved, and raped by members of ISIS (The Islamic State of Iraq and Syria) [8].

Even though “virginity testing” in all its forms has been condemned by human rights and international health organizations, it is still practiced in many countries around the globe, including in Afghanistan, Brazil, Egypt, India, Indonesia, Iran, Jamaica, Jordan, Palestine, South Africa, Swaziland, Turkey, and Zimbabwe, among others [7]. Increasingly, reports of this practice have been cited in some communities in countries such as Canada, Sweden, Spain and the Netherlands [5]. Virginity testing is a long-standing tradition in many parts of the world, not necessarily deemed illegal, and sometimes practiced by physicians. The exam itself can be painful and psychologically distressing to the women subjected to it [6]. Moreover, results of an examination of the hymen represent a means of influencing the way women and girls are viewed and treated in society based on related, though unsupported, conclusions about their veracity or believability, especially as it is related to their reported or purported sexual history. These factors have led all UN agencies to issue a call recently to ban this practice [7].

Despite an absence of evidence supporting the practice [9, 10], clinicians continue to refer to changes in the hymen to assess for a history of consensual or nonconsensual sexual intercourse. The authors of this manuscript have been involved in forensic sexual violence training of clinicians from various settings, primarily in East and Central Africa, through Physicians for Human Rights’ Program on Sexual Violence in Conflict Zones. We have noticed, anecdotally yet persistently, that many clinicians hold misconceptions about hymenal anatomy, morphology and physiology and the role the hymen can play in determining sexual activity – consensual or nonconsensual – particularly among children, adolescents, and young women.

Some clinicians, for example, reflecting common beliefs in many parts of the world [11], believe – inaccurately – that sexual acts will always result in changes to the hymen or that a “broken” hymen can be diagnostic of, and must serve as proof of sexual assault or rape. During the course of dozens of training workshops and medical chart reviews over more than 6 years, we have noted forensic medical evaluations characterizing the hymen as “broken”, “old and torn,” “intact,” “virginal,” or “dirty.” These misrepresentations, in our experience, are not unique to individuals from the medical sector, but have also been documented and prioritized as evidence among members of other sectors, including law enforcement officers involved in sexual assault investigations, lawyers and prosecuting magistrates involved in prosecutions, and judges responsible for adjudicating these cases.

Though other authors have addressed the unreliability of using hymen morphology to determine sexual history, we have found very few articles in recently published peer-reviewed literature that summarize misconceptions about the hymen [9]. Therefore, the purpose of this article is to critically review published evidence to dispel common myths about the hymen, its morphology, function, and its use as evidence in cases of sexual violence. We conducted a narrative literature review on PubMed that included the words: hymen (and morphology, anatomy), AND sexual violence; sexual assault; rape; sexual activity; sexual abuse, injury; intercourse. Other inclusion criteria: English language articles published between 1990 – September 2018.

Our field experience providing training to hundreds of clinicians through Physicians for Human Rights’ Program on Sexual Violence in Conflict Zones in the Central African Republic, Democratic Republic of the Congo (DRC), Iraq, and Kenya, guided our selection of the highlighted myths.

## Commonly encountered myths involving the hymen

### Myth #1: the hymen is a membranous tissue that completely covers the vaginal orifice

#### FACT 1A: the hymen is a membranous tissue that surrounds the vaginal orifice

The hymen is a membrane at the opening of the vagina. In early fetal life, the vagina is first formed as a solid tube. Over time, the inner portion of the tube disintegrates, so it becomes a hollow tubular structure. At the lower end of the tube a thin membrane, the hymen, typically remains. Often, this membrane ruptures in the first few days of life; it may remain as a rim of membrane around the vaginal orifice, or it may remain as a membrane with one or more small openings or rupture sites that partially cover the vagina. The size is variable and measurement depends on the position of the examination, though the diameter is generally described as smaller than 6 mm in prepubertal girls [10].

The vaginal canal extends internally from the hymen to the cervix. It is rare that the hymen covers the vaginal orifice completely (“imperforate hymen”) [12, 13], and the reported prevalence of this condition varies from approximately 1 case per 1000 population to 1 case per 10,000 population [13].

The hymen’s appearance can also be distinguished by the presence of polyps, tags, ridges, bands, and notches, and there is no standardized hymeneal appearance for young girls, adolescents, or adult women. Different configurations of the hymen exist, and include, most commonly: annular (also known as circumferential), crescentic, and fimbriated (with finger-like projections) [12, 14, 15].

Less common hymen configurations are: sleeve-like, septate (in which there are two openings with a band of tissue between them), cribiform (with multiple openings), micro-perforate (in which the hymenal orifice is extremely small), and imperforate (in which there is no hymenal opening at all). An imperforate hymen is often discovered at puberty when a patient presents with amenorrhea and hematocolpos [12, 13].

While at least two studies dating back to the 1980s and 1990s have reported hymens to be present in all newborn girls examined [1131 cases in one study and 134 in another], there are documented cases of girls born without a hymen altogether [16, 17]. In these cases, other genetic abnormalities, such as renal agenesis, were found. Large, cross-cultural, population-based studies that address the absence or presence of a hymen at birth have not been published. None of the existing studies provide or identify a clear function for the hymen [3, 4].

#### FACT 1B: the appearance of the hymen changes with age

The shape, size, and flexibility of the hymen vary, and change significantly, across a woman’s lifespan. In newborn babies, still under the influence of their mother’s hormones, the hymen is thick, pale pink, and redundant (folds in on itself and may protrude). For the first two to 4 years of life, the infant produces hormones that continue this effect. Over the next three to 4 years, the hymen changes and becomes the relatively thin, smooth-edged membrane that is usually associated with prepubertal female genitalia.

As puberty approaches, the hymen thickens, may assume a fimbriated or crescenteric appearance, and hymenal elasticity increases. Further changes occur with the hormonal changes of pregnancy, childbirth, aging, and the loss of hormonal production in menopause [18–20].

In summary, similar to other body parts and organs, hymeneal anatomy is extremely diverse and dynamic and it is imperative that those involved in routine and forensic gynecological examinations are aware of these wide variations and the scientific limits of what they may indicate.

## Myth #2: the presence or absence of hymenal tissue can be used to determine if a girl or woman has ever had sexual intercourse

### FACT: changes to the hymenal tissue’s anatomy are NOT necessarily indicative of having had intercourse (consensual or nonconsensual)

In cultures in which female virginity before marriage is prized, commonly assumed indicators of virginity are an “intact” hymen and blood on the sheets of the marital bed at first intercourse as a result of the hymen being “broken.” Multiple medical and scientific studies have refuted these assumptions and demonstrated that there is no evidence to support these beliefs [21–23]. In most cases, there is no correlation between a hymen’s appearance and the reported history of prior sexual intercourse.

#### Bleeding

The hymen is a membrane with relatively few blood vessels that – even if torn – may not bleed significantly. Forced penetration and lack of lubrication may cause lacerations to the vaginal wall, both of which are most likely to be responsible for the “blood-stained bed sheets,” rather than trauma to the hymen [21–23]. In fact, several studies have documented that bleeding is not routinely observed after a woman’s first sexual intercourse [21–23].

#### “Breaking” the hymen

In prepubertal girls, the hymen and vagina are smaller and less elastic than in adolescent and adult women, and consequently trauma due to penetration is more likely to be evident and more characteristic [24, 25]. However, studies have shown that physical evidence of penetration is generally lacking in most reported cases of initial consensual or nonconsensual sexual intercourse [26], even among prepubertal girls.

In postpubertal women, or at the beginning of their sexual life, the hymen may stretch, allowing vaginal penetration with minimal or no injury. Only a small portion of these women will exhibit changes in the hymen indicative of penetrating trauma. For example, in one small study of 36 pregnant adolescent girls, medical staff were only able to make definitive findings of penetration in two cases [27].

Another study comparing hymenal morphology in adolescent girls with and without a history of consensual sexual intercourse found that 52% of those who admitted to having had prior intercourse, had no identifiable changes to the hymenal tissue [28]. Similarly, where the morphology of the hymen has been altered, this can be attributed to causes other than sexual intercourse, including the insertion of objects, fingers, penetrating accidental trauma, and surgical procedures [29–31].

## Myth #3: vaginal examination of the hymen can determine whether sexual assault (specifically, nonconsensual penetration) has occurred

### FACT: alterations of the hymenal appearance are non-specific and, without corroboration with history and/or other forms of evidence, no medical or legal conclusion may be inferred by hymen examination alone

Hymenal measurements of size and width, lacerations and transection have been shown to lack specificity or sensitivity to confirm previous vaginal penetration. It is well recognized that similarities exist between naturally occurring variations and hymenal changes resulting from injury [12, 25, 32]. Even in children with suspected sexual abuse, the majority will have normal or nonspecific findings. Unless there are extensive laceration(s), hymenal injuries heal rapidly and usually leave no evidence of any previous injury [12].

A significant body of scientific evidence demonstrates that the vast majority of children who have been sexually abused, including with vaginal and anal penetration, have normal ano-genital examinations [12, 25, 33–37]. A study of 2384 children indicated that only 4% of children referred for a medical examination with a history of sexual abuse had abnormalities on physical exam. Similarly, a survey of pediatric child abuse rape cases indicated that only 2.1% of subjects examined had visible lesions on the hymen [36].

Studies of sexual assault survivors also provide evidence that the hymen may not incur noticeable damage as a result of forced penetration. In one study, only 19% of victims between the ages of 14 and 19 years – who identified as not having had prior sexual intercourse before the alleged sexual assault – had acute hymenal tears [37–39]. Another study involving a greater range of ages of women alleging sexual assault found that only 9.1% had hymenal perforation [40]. The authors of this study concluded that a substantial proportion of women, regardless of prior sexual experience, would not have visible genital injuries following forced vaginal penetration.

Therefore a “normal” examination of the genitals and anus neither confirms nor rules out sexual abuse. [41].

Additionally, some clinicians have been taught to measure the size and width of the hymenal orifice as part of the examination. However, in prepubertal girls, measurement of the hymenal orifice diameter or of the width of the hymen are of no value in diagnosing penetration due to the difficulties in obtaining a measurement, which varies with the examination position, technique, age of the child, state of relaxation of the child, and the skill of the examiner. Further, the appearance of the hymenal rim may change with examination position or technique [42]. Several experts and professional organizations, including the Royal College of Pediatrics and Child Health, and the U.S. National Protocol for Sexual Abuse Medical Forensic Examinations recommend against measurement of the hymenal orifice or the hymenal width. [41, 43].

One noticeable exception may be in the consideration of sexual abuse in prepubescent females [44, 45]. In this age group, penetrative abuse should at least be considered where there is complete or almost complete absence of posterior hymenal tissue (the area between 03.00 and 09.00 on a clock face, with the patient lying on their back).

Ultimately, evaluation of the hymen tissue, if visible, in and of itself, without supportive history, physical examination, or other forensic findings, could never answer the question of whether an individual – child or adult – had consensual or nonconsensual sex. Despite the lack of specificity and sensitivity, where sexual assault or abuse is alleged, a full forensic examination of the child or adult is imperative [46]. Table 1 summarizes some anatomical changes and signs and their potential etiologies and association with sexual activity.

**Table 1:** Summarizes some anatomical changes and signs and their potential etiologies and association with sexual activity

Type of anatomical findings	Description	Relevance to assessment of sexual assault
Lacerations	A laceration is defined as an acute tear through the full thickness of the skin or other tissues. When acute, a laceration may be associated with bleeding and /or bruising of the edges of the wound. A laceration to the hymen may involve the tearing of the full width of the hymen or only the partial width.	Though laceration of the hymen may indicate vaginal penetration, in one study of 205 prepubertal girls with confirmed history of vaginal penetration, only 33% had reported hymenal lacerations. [42, 48] Even though it is not common, a laceration of the hymen of any depth is highly suggestive of child sexual abuse. (68)
		Lacerations of the hymen have also been described, though rarely, following accidents. These injuries can mimic those seen in child sexual abuse. [47]
		In pubertal girls, evidence from five studies show that hymenal lacerations are seen in 3% to 19% of those who allege sexual abuse including penile penetration. [47]
Hymenal Transections	A transection is a defect in the posterior hymen rim that extends to or through the base of the hymen. A transection is not an acute injury, but it can be considered a sign of a healed injury.	Hymenal transections are very rarely seen in prepubertal girls who have not been sexually abused. However, a demonstrated transection, based on multiple studies, is commonly viewed as “a clear but uncommon indicator of past trauma” [42].
		A transection below the 3 to 9 o’clock location is the only non-acute hymen finding that is considered clear evidence of past injury (68). When hymenal transection are found, previous or past penetrative injury should be strongly suspected. (21, 68)
Bumps and Munds	A bump or a mund is a solid, localized, rounded, thickened area of tissue on the edge of the hymen.	Bumps and mounds are considered a normal variant. They have been frequently observed in both abused and non-abused prepubertal girls. Several investigators found bumps in the anterior half of the hymen and in the posterior half of the hymen with similar frequency in abused and non-abused prepubertal girls. [42].
Clefts and Notches	Cleft and notches are indentations in the hymenal membrane.	Clefts and notches in the anterior hymen have been described in newborn and in prepubertal sexually abused and non-abused girls.
	Clefts and notches can be part of the normal hymenal morphology in a fimbriated hymen. Superficial notches are defined as a notch inferior to 50% of the width of the hymenal membrane.	
		Superficial notches (inferior to 50%) have been reported in both prepubertal girls with a history of vaginal penetration and prepubertal girls selected for non-abuse.
		Deep clefts or notches in the posterior half of a non-fimbriated hymen have only been reported in prepubertal girls with a history of vaginal penetration.
		In pubertal girls, posterior deep notches or complete clefts (transections) have been reported more often in girls with a history of forced (nonconsensual) vaginal penetration or consensual sexual intercourse than in girls denying sexual intercourse (33% vs 7%) [49]
		The finding of hymenal clefts increased with age. [48]
Scars		Hymenal lacerations can heal completely without scarring. They may also heal to leave a notch or a full transection. [47]

## Myth #4: clinicians are well trained to identify the morphology and physiology of the hymen and to draw conclusions based on their inspection and examination

### Fact: clinicians receive little training in or exposure to the assessment of hymen morphology

Even for experienced physicians, it may be extremely difficult to differentiate between lacerations or other changes resulting from vaginal penetration and naturally occurring morphological changes [47, 48].

Assumptions about normal variations are common among non-specialized practitioners, many of whom mistakenly believe that normal variations indicate a history of sexual abuse [49]. Normal variants, commonly mistaken as signs of sexual trauma or a history of sexual abuse, include anal fissures, genital nevi, genital erythema, enlargement of the hymenal opening, failure of midline fusion of the hymen, narrowing of the hymenal edge, partial hymenal notching, hymenal clefts, and even conditions such as lichen sclerosis [50].

One study lamented that “Pediatricians … demonstrate knowledge gaps about the hymen” [51]. A report documenting the case of a 14-year-old in Turkey who was evaluated for sexual abuse noted that “The girl was sent for hymen examination three times because two gynecologists and one forensic expert produced different witness reports, confusing the court.” [48].

This is not surprising, considering that during their medical education, medical students receive only a few hours of pelvic examination training. Because medical students’ exposure to pelvic exam training involves mostly adult volunteers and standardized patients, as well as pelvic models, there are very few opportunities to teach and learn about the hymen in medical school.

Many medical learners report high levels of discomfort performing genital exams [52–55], though simulation and working with standardized patient volunteers appear to lessen their discomfort. Pelvic exam competencies tend to focus on performing a vaginal and bimanual exam, as well as a pap smears. Manuals for clinical teachers of the adult female pelvic exam do specify “evaluation of the introitus,” but the hymen is rarely mentioned explicitly [56, 57]. These studies are primarily in the context of medical education in English speaking countries. A study surveying medical students in Saudi Arabia [58] found that “most student never performed” a physical examination of an “intimate area” on females or males and over 40% had never taken a sexual history during their course. In another study from Saudi Arabia, 43% of survey students never performed a female pelvic examination [59]. This is likely no different in other countries, although there are no specific articles addressing this issue in other contexts.

The situation is a bit different when it comes to resident teaching in the U.S. context, where the hymen is mentioned explicitly, but only in some curricula, for general internal medicine residents, [60] pediatric residents [61], and for pediatric gynecology trainees. [62, 63].

Complicating this issue are ethical considerations, especially when it comes to the genital examination of children. One cannot ethically subject anyone (child, adolescent, or adult) to a genital examination simply for the purpose of learning about human anatomy and physiology (as one would, for example, a cardiac exam). Opportunities to examine the hymen may be rare and coincident with specific – and uncommon – indications, severely limiting the number of opportunities for observation, examination, and assessment of the hymen.

## Summary

There is no evidence that examination of the hymen is an accurate or reliable test of a previous history of sexual activity, including sexual assault. As discussed, there are many factors that confound whether clinicians can adequately assess changes to the hymen tissue at various stages of the life cycle, including genetic, developmental, endocrine, spontaneous, and external influences.

Information gleaned from a forensic assessment that includes an ano-genital examination can support a legal charge of various forms of sexual violence. However, it is important to note that several types of forensic evidence are needed to support a legal charge, including patient history, physical evidence (e.g. injuries to the genital and non-genital regions of the body), psychological evidence, laboratory evidence (e.g. spermatozoa, DNA, and sexually transmitted organisms), or other forms of forensic evidence. During the physical examination, the hymen tissue may or may not be present, or be present with or without abnormalities, depending on the patient’s age, history, and other factors.

A reliance on examination of the hymen to determine sexual history in general may result in individual and societal harms, including psychological sequelae associated with mistrust and disrespect for bodily sovereignty, physical discomfort and pain, in addition to the possibility of drawing inaccurate conclusions about sexual violence.

**Fig. 1:**
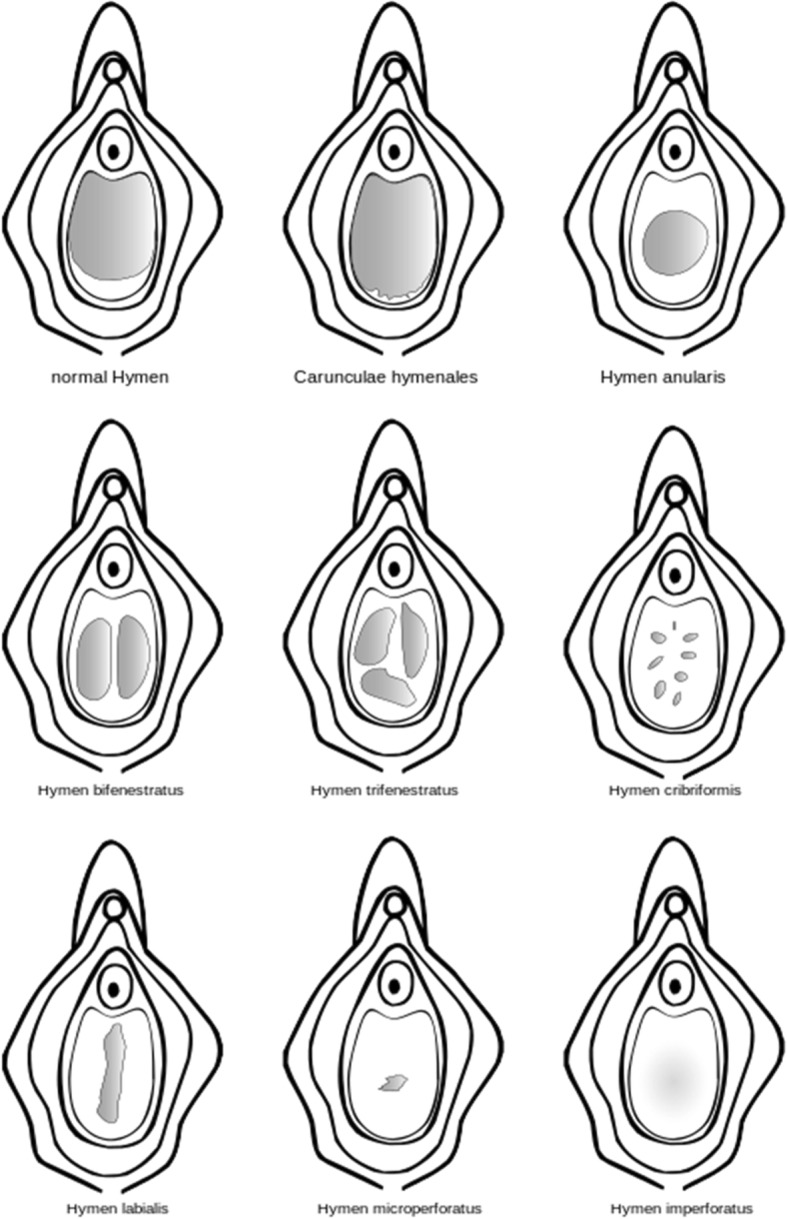
Shows some of the different configurations of the hymen

**Fig. 2:**
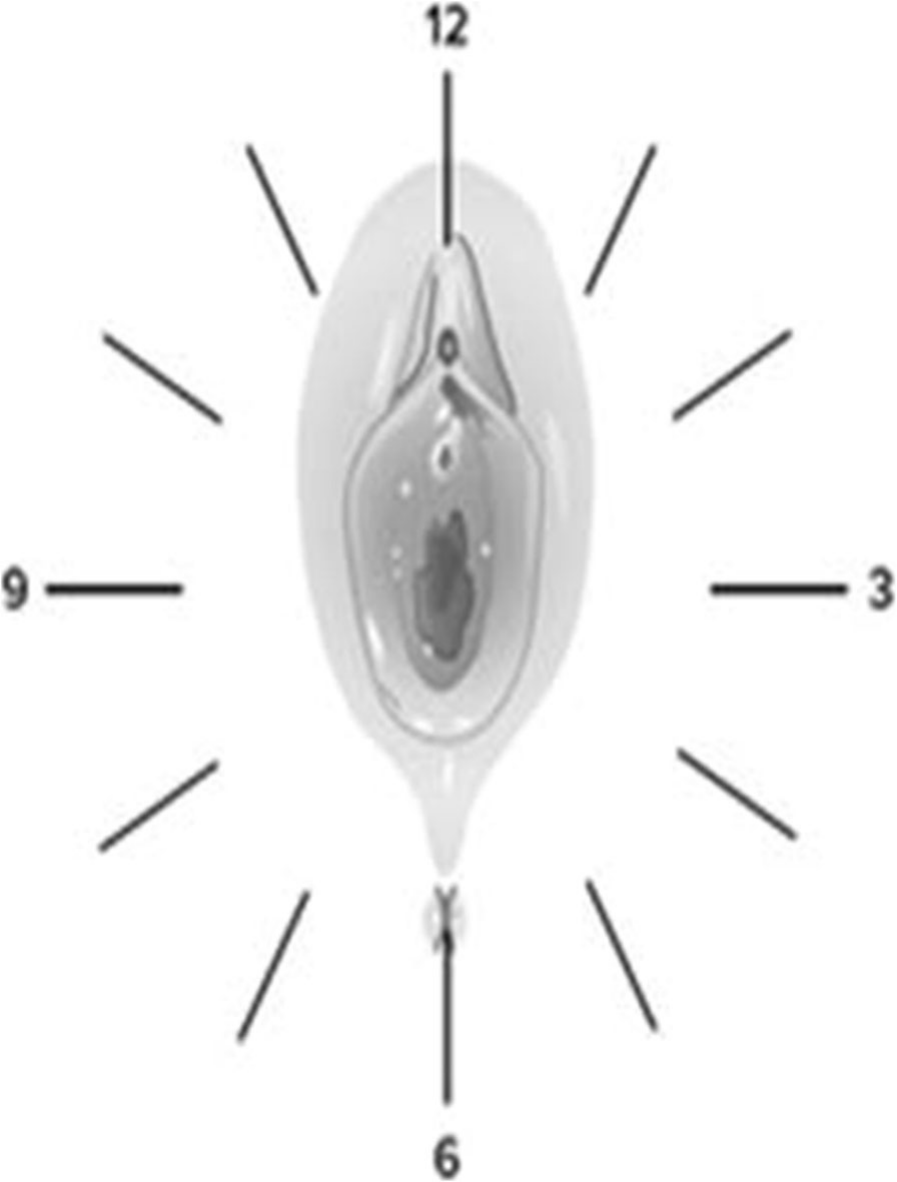
Suggests a visual representation of the introitus for consistent description in clinical reporting

Clinicians tasked with performing forensic sexual assault examinations should be aware that there is no gold standard for a “normal” hymen, nor is there one for conducting any sort of diagnostic or predictive hymen examination. Descriptions such as “intact hymen” or “broken hymen” should be avoided in all cases. Clinicians should describe specific findings using international standards and terminology of morphological features (See Figs. 1 and 2).

Given that hymen examinations rarely lead to a determination of whether the hymen or vagina was penetrated by a penis or other object, they have little to no diagnostic or forensic value. We call on clinicians to consider the very low predictive value of a hymen examination and to: 1) avoid relying solely on the status of the hymen in sexual assault examinations and reporting; 2) help raise awareness of this issue among their colleagues and counterparts in law enforcement and the judiciary; and 3) promote a discussion of the limitations of examining the hymen as part of clinical education for all specialties engaged in sexual or reproductive healthcare of women and girls.

Additional file 1 contains a French version of this original article.

## Additional file

Additional file 1: Translation of this article into French. (DOCX 91 kb)
