# Supplementary material for: The little tissue that couldn’t – dispelling myths about the Hymen’s role in determining sexual history and assault
Source: Reprod Health. 2019 Jun 3;16:74. doi: 10.1186/s12978-019-0731-8 (PMC6547601; doi:10.1186/s12978-019-0731-8)
Supplement: Supplementary file 1 — Translation of this article into French. (DOCX 91 kb) [file 12978_2019_731_MOESM1_ESM.docx]

**Cette petite membrane et ses limites: réfuter les mythes sur la capacité de l’hymen à témoigner de l’activité sexuelle ou des agressions sexuelles**

Ranit Mishori, MD, MHS, FAAFP, Hope Ferdowsian, MD, MPH, FACP, FACPM, Karen Naimer, JD, LLM, MA, Muriel Volpellier, MD, MFFLM, MPH, Thomas McHale, SM.

1. Ranit Mishori, MD, MHS, FAAFP Department of Family Medicine, Georgetown University, Ecole de médecine, Washington, DC. USA [mishorir@georgetown.edu](mailto:mishorir@georgetown.edu)
2. Hope Ferdowsian, MD, MPH, FACP, FACPM Department of Medicine, University of New Mexico, École de médecine, Albuquerque, NM, USA hopeferdowsian@gmail.com
3. Karen Naimer, JD, LLM, MA. Physicians for Human Rights, Directrice de Programme sur les violences sexuelles en zones de conflit, Boston, Massachusetts. [knaimer@phr.org](mailto:knaimer@phr.org)
4. Muriel Volpellier, MD, MFFLM, MPH Sexual Assault Referral Centre, the Haven Paddington, Kings College Hospital NHS Trust London [murielvolpellier@doctors.org.uk](mailto:murielvolpellier@doctors.org.uk)
5. Thomas McHale, SM. Physicians for Human Rights, Spécialiste Principal de Programme sur les violences sexuelles en zones de conflit, Boston, Massachusetts tmchale@phr.org

Auteur :

Ranit Mishori, MD, MHS, FAAFP
Professeur de médecine

Georgetown University, École de médecine

3900 Reservoir Rd. NW

Washington, DC, 20007

[mishorir@georgetown.edu](mailto:mishorir@georgetown.edu)

Word Count 3,167

Tables: 1

Figures: 2

**Cette petite membrane et ses limites : réfuter les mythes sur la capacité de l’hymen à témoigner de l’activité sexuelle ou des agressions sexuelles**

Word Count 3,167

Tables: 1

Figures: 2

Abstract (221 words)

Dans certains cas, des conclusions sur la vie sexuelle des femmes et des filles sont basées sur des hypothèses concernant l’hymen, un petit tissu membraneux sans fonction biologique connue, qui occupe une partie de l’ouverture vaginale externe de la femme. Les cliniciens continuent cependant à se référer à des changements de l’hymen pour évaluer si des rapports sexuels, consentis ou forcés, ont eu lieu. Nous avons étudié les données qui ont été publiées dans le but de réfuter les mythes communément admis sur l’hymen et sa morphologie, sa fonction, et son utilisation comme élément de preuve dans les affaires de violences sexuelles.

L’examen de l’hymen ne constitue pas une indication précise ou fiable de l’activité sexuelle passée, y compris en matière d’agression sexuelle. Les cliniciens chargés de procéder à un examen médico-légal spécifique aux agressions sexuelles, devraient systématiquement éviter le recours à des termes comme « hymen intact » ou « hymen déchiré » et devraient donner leurs conclusions de manière précise, en utilisant la terminologie et les normes internationales des caractéristiques morphologiques.

Nous appelons les cliniciens à tenir compte de la faible valeur prédictive de l’examen de l’hymen et : 1) éviter de s’en remettre uniquement aux caractéristiques de l’hymen dans les examens et les comptes rendus en matière d’agression sexuelle 2) faciliter la prise de conscience de ce problème chez leurs homologues et partenaires des forces de l’ordre et du système judiciaire ; et 3) encourager, au cours de la formation clinique de tous les spécialistes impliqués dans la santé sexuelle ou reproductive des femmes et des filles, les discussions factuelles sur les limites de l’examen de l’hymen.

Plain English Summary (234 words)

Dans certains cas, les cliniciens qui examinent les femmes et les filles présumées victimes d’agression à caractère sexuel ou suspectées d’avoir eu des rapports sexuels (consentis ou non) fondent leur évaluation sur l’examen de l’hymen. L’hymen est un petit tissu membraneux situé à l’extérieur du canal vaginal, sans fonction biologique connue. Nous nous sommes penchés sur les études publiées concernant l’hymen afin de nous aider à conseiller les cliniciens sur la pertinence de procéder à un examen de l’hymen et dans quel cas, le réaliser.

Nous avons conclu que cet examen ne constitue pas une indication précise ou fiable de l’activité sexuelle, y compris dans les cas d’agression sexuelle, sauf dans des situations très particulières. Les cliniciens qui procèdent à un examen médico-légal spécifique aux agressions sexuelles, devraient systématiquement éviter le recours aux descriptions de type « hymen intact » ou « hymen déchiré » et décrire leurs conclusions cliniques, en ayant recours à la terminologie médicale.

Nous conseillons aux cliniciens de tenir compte du fait que l’examen de l’hymen n’offre généralement pas un degré élevé de certitude sur l’activité sexuelle consentie ou non consentie. Nous appelons les cliniciens à : 1) éviter de s’en remettre uniquement aux caractéristiques de l’hymen dans les examens et les comptes rendus en matière d’agression sexuelle 2) faciliter la prise de conscience de ce problème chez leurs homologues et partenaires des forces de l’ordre et du système judiciaire ; et 3) encourager les discussions factuelles sur les limites des examens de l’hymen avec leurs collègues et avec les étudiants en médecine, dans toutes les spécialités en lien avec la santé sexuelle ou reproductive des femmes et des filles.

LE CONTEXTE

Dans de nombreuses cultures patriarcales, la vie sexuelle des filles et des femmes est importante lorsqu’il s’agit de déterminer leur statut au sein de la société, de la communauté, de la famille, et également, en tant que personne (1, 2). Fréquemment, des conclusions sur la vie sexuelle sont basées sur des hypothèses concernant l’hymen, un petit tissu membraneux sans fonction biologique connue (3, 4) qui occupe généralement une partie de l’ouverture vaginale externe chez les femmes.

Dans certaines communautés, la signification socioculturelle de l’hymen, comme « preuve » de l’absence d’activité sexuelle, entremêle culture, religion, politique, droit, économie et médecine et prend la forme d’un « test de virginité » qui se traduit par un examen vaginal visant à évaluer si l’hymen d’une femme est « intact » et tentant d’établir si elle a eu des rapports sexuels (5,6). C’est une pratique courante avant le mariage. Les évaluations sont souvent menées par des prestataires de soins qui déterminent, par le biais de ces examens (6,7), si une femme ou une fille a eu des rapports sexuels.

Dans certaines régions du monde, les filles et les femmes ont été soumises à un examen de l’hymen pour prouver qu’elles ont subi des violences sexuelles, comme dans le cas des femmes yézidies qui furent kidnappées, asservies, et violées par des membres del’État Islamique en Irak et en Syrie (8).

Bien que toutes les formes de « test de virginité » aient été condamnées par les organisations internationales intervenant dans le domaine de la santé ou des droits humains, cette pratique persiste dans de nombreux pays du globe, notamment en Afghanistan, au Brésil, en Égypte, en Inde, en Indonésie, en Iran, en Jamaïque, en Jordanie, en Palestine, en Afrique du Sud, au Swaziland, en Turquie et au Zimbabwe (7). Cette pratique est également de plus en plus signalée dans certaines communautés établies dans des pays comme le Canada, la Suède, l’Espagne et les Pays-Bas (5). Le test de virginité est une tradition ancienne dans de nombreuses régions du monde, pas nécessairement illégal et parfois pratiqué par des médecins. L’examen en lui-même peut être douloureux et déstabilisant d’un point de vue psychologique pour les femmes qui y sont soumises (6). De plus, l’examen de l’hymen peut, de par ses résultats, constituer un moyen d’influencer la perception et le traitement que la société porte sur les femmes et les filles, sur leur honnêteté ou leur crédibilité, en particulier en lien avec ce qu’elles disent de leur vie sexuelle et ce, sur la base de conclusions sans fondement. Ces éléments ont amené l’ensemble des agences onusiennes à lancer récemment un appel en faveur de l’interdiction de cette pratique (7).

Malgré l’absence de données tangibles corroborant cette pratique (9, 10), les cliniciens continuent à se référer aux modifications de l’hymen pour évaluer si des rapports sexuels consentis ou forcés ont eu lieu. Les auteurs de ce texte ont participé, via le Programme sur les violences sexuelles en zones de conflit de Physicians for Human Rights, à la formation médico-légale, dans le cadre des violences sexuelles, de cliniciens de diverses zones géographiques, en premier lieu en Afrique de l’Est et en Afrique Centrale.

Nous avons remarqué que de nombreux cliniciens ont parfois des conceptions erronées sur l’anatomie, la morphologie et la physiologie de l’hymen ainsi que sur le rôle qu’il peut jouer pour déterminer l’activité sexuelle, consentie ou non, en particulier chez les enfants, les adolescentes et les jeunes femmes.

Par exemple, certains cliniciens, reflétant des croyances répandues dans de nombreuses régions du monde (11), pensent à tort, que les rapports sexuels entraînent systématiquement des modifications de l’hymen ou qu’un hymen « déchiré », peut servir à témoigner d’une agression sexuelle ou d’un viol et doit servir de preuve. Au cours d’une douzaine de sessions de formation et plus de six années à examiner des dossiers médicaux, nous avons remarqué que des évaluations médico-légales caractérisaient l’hymen à l’aide des termes « déchiré », « vieilli et détruit », « intact », « virginal », ou « sale ». Ces représentations erronées, d’après notre expérience, ne sont pas propres aux intervenants du secteur médical mais ont également été documentées et privilégiées comme éléments de preuve par des intervenants d’autres secteurs, notamment des officiers de police impliqués dans les enquêtes sur les agressions sexuelles, des avocats, des juges d’instruction et des magistrats en charge de statuer sur ces affaires.

Si d’autres auteurs ont abordé l’absence de fiabilité de l’utilisation de la morphologie de l’hymen comme témoin de l’activité sexuelle, nous n’avons trouvé que très peu d’informations spécialisées publiées récemment qui résument les conceptions erronées sur l’hymen (9). Par conséquent, le but de cet article est de procéder à un examen critique des données publiées afin de réfuter les mythes communément admis sur l’hymen, sa morphologie, sa fonction, et son utilisation en tant qu’élément de preuve dans les affaires de violences sexuelles. Nous avons cherché les informations existantes sur PubMed à l’aide des termes suivants : hymen (ainsi que morphologie et anatomie), violences sexuelles, agression sexuelle, viol, activité sexuelle, abus sexuels, blessure et rapports sexuels. Nous avons également utilisé le critère suivant : articles en anglais publiés entre 1990 et septembre 2018.

Notre expérience du terrain dans la formation de centaines de cliniciens via le Programme sur les violences sexuelles en zones de conflit de Physicians for Human Rights en République centrafricaine, en République démocratique du Congo, en Irak et au Kenya, nous a guidé dans notre choix des mythes à souligner.

MYTHES COURAMMENT RENCONTRÉS SUR L’HYMEN

Mythe #1 : L’hymen est un tissu membraneux qui couvre complètement l’orifice vaginal.

FAIT 1A : L’hymen est un tissu membraneux qui entoure l’orifice vaginal.

L’hymen est une membrane se trouvant à l’ouverture du vagin. Au début de la vie fœtale, le vagin prend initialement la forme d’un conduit tubulaire solide dont la partie intérieure, au fil du temps, se désintègre pour devenir une structure tubulaire creuse. Sur la partie inférieure du conduit tubulaire, une fine membrane, l’hymen, reste généralement en place. Souvent, cette membrane se rompt au cours des premiers jours de la vie. Les contours de cette membrane peuvent rester autour de l’orifice vaginal ou cette membrane peut rester avec une ou plusieurs petites ouvertures ou ruptures qui couvrent partiellement le vagin. Sa taille est variable et sa dimension dépend de la position du patient lors de l’examen, bien que son diamètre soit généralement décrit comme étant inférieur à 6 mm chez les filles prépubères (10).

Le canal vaginal va de l’hymen au col de l’utérus. Il est rare que l’hymen couvre complètement l’orifice vaginal (« hymen non perforé ») (12, 13) et la prévalence signalée de cette condition varie d’environ 1 cas pour 1 000 à 1 cas pour 10 000 au sein de la population (13).

L’apparence de l’hymen peut également se distinguer par la présence de polypes, d’acrochordons, de caroncules, de bandes, et d’encoches, et il n’y a aucune apparence standard de l’hymen chez les jeunes filles, les adolescentes, ou les femmes adultes. L’hymen peut prendre différentes formes, notamment plus communément, la forme annulaire (également appelé circonférentielle), semi-lunaire ou fimbriée (12, 14, 15).

Moins communément, l’hymen peut, en apparence, être redondant, à pont (deux ouvertures avec une bande de tissu au centre), cribiforme (avec des ouvertures multiples), microperforé (avec un très petit orifice hyménal) et non perforé (sans aucune ouverture hyménale). Un hymen non perforé est souvent découvert lors de la puberté en présence d’une aménorrhée et d’hématocolpos chez un patient (12, 13).

Le schéma 1 montre certaines des différentes configurations que peut revêtir l’hymen.

Alors qu’au moins deux études datant des années 1980 et 1990 ont indiqué la présence de l’hymen chez la totalité des nouveau-nées examinés (1 131 cas dans une étude et 134 dans l’autre), des filles nées sans hymen ont également été répertoriées (16,17). Dans ce cas, d’autres anomalies génétiques, comme l’agénésie rénale, ont été constatées. Aucune étude démographique interculturelle, de grande ampleur, observant l’absence ou la présence de l’hymen à la naissance, n’a été publiée. Les études existantes n’attribuent pas de fonction clairement identifiée à l’hymen (3, 4).

FAIT 1B : L’apparence de l’hymen évolue avec l’âge.

L’hymen, dans sa forme, sa taille et son élasticité varie selon les femmes et évolue de manière significative au cours de leur vie. Chez les nouveau-nées, encore sous l’influence des [hormones](https://en.wikipedia.org/wiki/Hormones) de leur mère, l’hymen est épais, rose pâle et redondant (il se plie sur lui-même et peut former une protubérance). Au cours des deux à quatre première années de vie, le nourrisson produit des hormones qui prolongent cet effet. Lors des trois ou quatre années suivantes, l’hymen change et devient la membrane relativement fine, à bords lisses, qui est généralement associée aux organes génitaux féminin lors de la prépuberté.

A l’approche de la puberté, l’hymen s’épaissit et peut devenir fimbrié ou semi-lunaire et l’élasticité hyménale augmente. D’autres changements surviennent avec les changements hormonaux de la grossesse, de l’accouchement, du vieillissement et lors de la perte de production hormonale liée à la ménopause (18-20).

En résumé, tout comme d’autres parties et organes du corps humain, l’anatomie de l’hymen est extrêmement diverse et évolutive et il est impératif que ceux qui prennent part aux examens gynécologiques médico-légaux ou classiques, aient conscience de ces variations importantes et des limites scientifiques de leurs enseignements.

Mythe #2 : La présence ou l’absence de tissu hyménal peut être utilisée pour déterminer si une fille ou une femme a déjà eu des rapports sexuels.

FAIT : La modification de l’anatomie du tissu hyménal n’indique PAS nécessairement que des rapports sexuels (consentis ou forcés) aient eu lieu.

Dans les cultures qui attachent une grande importance à la virginité de la femme avant le mariage, la virginité est généralement associée à un hymen « intact » et à la présence de sang sur les draps du lit nuptial lors du premier rapport sexuel suite à l’hymen qui se « déchire ». Une multitude d’études médicales et scientifiques ont réfuté ces hypothèses et démontré qu’aucun élément de preuve ne soutient ces croyances (21-23). Dans la plupart des cas, il n’existe aucun lien entre l’apparence de l’hymen et l’antériorité de rapports sexuels.

Le saignement

L’hymen est une membrane ayant relativement peu de vaisseaux sanguins qui, même s’il est déchiré, peut ne pas saigner beaucoup. La pénétration forcée et le manque de lubrification peuvent causer des lacérations à la paroi vaginale et sont plus susceptibles d’entraîner des « tâches de sang sur les draps » qu’un traumatisme fragilisant l’hymen (21-23). En fait, plusieurs études ont montré que le saignement n’est pas couramment observé après le premier rapport sexuel d’une femme (21-23).

« Déchirer » l’hymen

Chez les filles prépubères, l’hymen et le vagin sont plus petits et moins élastiques que chez l’adolescente et la femme adulte, et le traumatisme causé par la pénétration est par conséquent plus susceptible d’être évident et caractéristique (24, 25). Cependant, des études ont montré que les preuves physiques de pénétration sont généralement absentes dans la plupart des cas signalés de rapports sexuels consentis ou forcés (26) et ce, même chez les filles prépubères.

Chez les femmes postpubertaires, ou au début de leur vie sexuelle, l’hymen peut s’étirer, permettant la pénétration vaginale avec absence ou quasi-absence de blessure. Seul un petit nombre de ces femmes va présenter des modifications de l’hymen indiquant un traumatisme par pénétration. Par exemple, dans une étude à petite échelle, menée sur 36 adolescentes enceintes, le personnel soignant a pu conclure à une pénétration uniquement dans deux cas (27).

Une autre étude comparant la morphologie hyménale chez les adolescentes ayant eu, ou n’ayant pas eu, de rapports sexuels consentis, a conclu que 52 pour cent de celles qui avaient admis avoir déjà eu des rapports sexuels, ne présentaient aucun changement identifiable du tissu hyménal (28). De même, lorsque la morphologie de l’hymen a été altérée, cela peut être attribué à d’autres raisons que les rapports sexuels, notamment l’insertion d’objets ou de doigts, ou à un traumatisme lié à une pénétration accidentelle ou à une intervention chirurgicale (29-31).

Mythe #3 : L’examen vaginal de l’hymen peut permettre d’attester de l’agression sexuelle (en particulier en cas de pénétration forcée).

FAIT : Les altérations de l’apparence hyménale ne sont pas spécifiques et en l’absence de corroboration par les antécédents ou d’autres types de preuves, aucune conclusion médicale ou légale ne peut être tirée par le seul examen de l’hymen.

Il a été démontré que la taille et la largeur de l’hymen, ainsi que les lacérations et les transections n’ont pas un degré de spécificité et de certitude suffisant pour attester d’une pénétration vaginale. Il est bien établi que des similarités existent entre les modifications de l’hymen dues à des causes naturelles et celles dues à une blessure (12, 25, 32). Même chez les enfants présumés victimes de violences sexuelles, la majorité d’entre eux ne présentent pas de signes spécifiques. A moins que les lacérations ne soient importantes, les blessures hyménales guérissent rapidement et ne laissent généralement aucun élément témoignant du traumatisme (12, 33-25).

De nombreuses preuves scientifiques démontrent que chez la grande majorité des enfants ayant subi des violences sexuelles, y compris en cas de pénétration vaginale et anale, l’examen anogénital ne révèle aucune anomalie (12, 25, 33-37). Une étude menée sur 2 384 enfants a montré que l’examen médical présentait des anomalies chez seulement 4 pour cent des enfants ayant subi des violences sexuelles. De même, une étude sur les cas de viol d’enfants a montré que les sujets examinés n’étaient que 2,1 pour cent à présenter des lésions visibles de l’hymen (36).

Les études menées sur les victimes d’agressions sexuelles donnent également des éléments tangibles sur le fait que l’hymen peut ne pas subir de dommages perceptibles en cas de pénétration forcée. Dans une étude, seulement 19 pour cent des victimes âgées de 14 à 19 ans, que l’on avait identifiées comme n’ayant pas eu de rapports sexuels avant l’agression sexuelle présumée, présentaient de sévères déchirures hyménales (37). Une autre étude avec une amplitude d’âge plus forte, sur des femmes victimes d’agression sexuelle, a conclu que, seulement 9,1 pour cent d’entre elles présentaient une perforation hyménale (40). Les auteurs de cette étude ont conclu qu’une proportion substantielle de femmes, quel que soit leur passé sexuel, ne présentait aucune lésion génitale visible suite à une pénétration vaginale forcée.

Par conséquent, un examen « normal » des organes génitaux et de l’anus ne permet pas de confirmer ou d’exclure les violences sexuelles. (41).

De plus, certains cliniciens ont reçu comme directive de mesurer la taille et la largeur de l’orifice hyménal dans le cadre de l’examen. Cependant, chez les filles prépubères, mesurer le diamètre de l’orifice hyménal, ou la largeur de l’hymen, n’est d’aucune valeur pour attester une pénétration, en raison de la difficulté de mesurer une dimension qui varie en fonction de la position du patient pendant l’examen, de la technique, de l’âge de l’enfant, de son état de relaxation et des compétences de la personne procédant à l’examen. Enfin, l’apparence du contour hyménal peut changer, selon la position du patient pendant l’examen ou la technique (42). Plusieurs experts et organisations professionnelles, notamment le Royal College of Pediatrics and Child Health et le U.S. National Protocol for Sexual Abuse Medical Forensic Examinations ont recommandé de ne pas mesurer l’orifice hyménal ou la largeur de l’hymen. (41, 43)

Le tableau 1 résume certains changements et signes anatomiques, leurs causes éventuelles et le lien avec l’activité sexuelle.

Une exception notable pourrait concerner les violences sexuelles chez les femmes prépubères (44, 45). Dans ce groupe d’âge, la pénétration forcée devrait être prise en considération en cas d’absence totale ou quasiment totale de tissu hyménal postérieur (la zone entre 3 et 9 heures selon les aiguilles d’une montre, avec le patient couché sur le dos).

Le schéma 2 suggère une représentation visuelle de l’hymen pour assurer la cohérence des descriptions employées dans les rapports cliniques.

Au final, l’évaluation du tissu de l’hymen, si celui-ci est visible, en l’absence des antécédents du patient, d’examen physique ou d’autres conclusions médico-légales, ne peut jamais répondre à la question consistant à savoir si une personne, qu’elle soit un enfant ou d’âge adulte, a eu des rapports sexuels consentis ou forcés. Malgré son faible degré de spécificité et de certitude, en cas d’agression sexuelle présumée, un examen médico-légal complet de l’enfant ou de l’adulte est impératif (46).

Mythe #4 : Les cliniciens ont reçu une formation solide leur permettant d’identifier la morphologie et la physiologie de l’hymen et de tirer des conclusions fondées sur le contrôle médical et les examens auxquels ils procèdent.

Fait : Les cliniciens ne sont que peu formés ou exposés à l’évaluation de la morphologie de l’hymen.

Même pour les médecins expérimentés, il peut être extrêmement difficile de différentier les lacérations ou autres changements résultant de la pénétration vaginale des changements morphologiques naturels (47, 48).

Les hypothèses sur les variations normales sont courantes chez les praticiens non spécialisés qui sont nombreux à penser, à tort, que ces dernières indiquent des antécédents de violence sexuelle (49). Les variations normales, couramment interprétées, à tort, comme des signes de traumatisme sexuel ou de violence sexuelle, incluent les fissures anales, les nævus génitaux, l’érythème génital, l’agrandissement de l’ouverture hyménale, les anomalies de fusion de la ligne médiane de l’hymen, le rétrécissement du bord de l’hymen, les encoches partielles de l’hymen, les fissures hyménales et même des pathologies comme le lichen scléreux (50).

Une étude a déploré que les « pédiatres … montrent des lacunes en matière de connaissance de l’hymen » (51). Un rapport relatant le cas, en Turquie, d’une fille âgée de 14 ans, présumée victime d’agression sexuelle, note que « la fille a passé trois examens de l’hymen car deux gynécologues et un expert médico-légal avaient présenté des rapports d’expertises différents qui avaient dérouté le tribunal ». (52).

Ce n’est pas surprenant car, au cours de leur cursus, les étudiants en médecine ne suivent que quelques heures de formation sur l’examen pelvien, se limitant principalement à des adultes volontaires, des patients types et des modèles pelviens. Ainsi, les opportunités d’enseignement et d’apprentissage sur l’hymen sont très limités dans les écoles de médecine.

Les étudiants en médecine sont nombreux à dire que procéder à un examen génital leur est particulièrement inconfortable (53, 54, 55, 56) bien que les simulations, et le fait de travailler avec des patients types volontaires, semblent diminuer ce niveau d’inconfort. Les compétences en matière d’examen pelvien ont tendance à se concentrer sur la réalisation d’un examen vaginal et bi-manuel ainsi qu’un frotti cervico-utérin. Les manuels à destination du personnel enseignant parlent de « l’évaluation du vagin » pour ce qui est de l’examen pelvien des femmes adultes mais l’hymen est rarement mentionné de manière explicite (57, 58). Ce sujet est principalement abordé dans le cadre de l’éducation médicale des pays anglophones. Une étude sur les étudiants en médecine en Arabie Saoudite (59) a conclu que « pour la plupart, les étudiants n’avaient jamais procédé » à un examen physique d’une « région intime » chez les femmes ou les hommes et plus de 40 % d’entre eux n’avait jamais relevé les antécédents sexuels d’un patient pendant leur formation. Dans une autre étude sur l’Arabie Saoudite, 43 % des étudiants interrogés n’avaient jamais procédé à l’examen pelvien d’une patiente (60). La situation est probablement similaire dans d’autres pays bien que cela ne fasse l’objet d’aucun article spécifique.

La situation est un peu différente pour ce qui est de l’enseignement aux résidents en médecine aux États-Unis, où l’hymen est mentionné de manière explicite, mais seulement dans certains cursus, pour les résidents de médecine interne générale (61), les résidents en pédiatrie (62) et les stagiaires en gynécologie pédiatrique. (63, 64).

Les considérations éthiques compliquent cette question, en particulier s’agissant de l’examen gynécologique des enfants. D’un point de vue éthique, personne (enfant, adolescent ou adulte) ne peut passer un examen gynécologique dans une simple finalité d’apprentissage de l’anatomie humaine et de la physiologie (comme cela pourrait être le cas, par exemple, pour un examen cardiaque). Les occasions d’examiner l’hymen peuvent être rares et coïncident avec des indications spécifiques et peu fréquentes, limitant grandement les opportunités d’observer, d’examiner et d’évaluer l’hymen.

RÉSUMÉ

Aucun élément de preuve ne va dans le sens de la précision ou de la fiabilité de l’examen de l’hymen pour identifier les antécédents sexuels et notamment, pour attester d’une agression sexuelle. Comme nous l’avons vu précédemment, nombreux sont les facteurs (génétiques, développementaux, endocriniens, impromptus et externes) qui réfutent la possibilité pour les cliniciens, d’évaluer de manière appropriée, les modifications du tissu de l’hymen à différentes étapes du cycle de la vie.

Les informations collectées lors d’une évaluation médico-légale comprenant un examen anogénital, peuvent appuyer les éléments à charge dans le cadre de poursuites judiciaires pour diverses formes de violences sexuelles. Cependant, il est important de noter que plusieurs types de preuves médico-légales sont nécessaires pour appuyer les éléments à charge, notamment les antécédents du patient, les preuves physiques (par exemple, les blessures aux parties génitales et non génitales du corps), les éléments psychologiques, les données de laboratoire (par exemple, les spermatozoïdes, l’ADN et les organismes transmis sexuellement) ou d’autres formes de preuves médico-légales. Au cours de l’examen médical, le tissu de l’hymen peut être présent ou ne pas l’être. Il peut également présenter des anomalies ou cela peut ne pas être le cas, en fonction de l’âge du patient, de ses antécédents, et d’autres facteurs.

S’en remettre à l’examen de l’hymen pour identifier l’activité sexuelle, peut entraîner des conséquences négatives sur le plan personnel et sociétal, notamment des séquelles psychologiques liées à la suspicion et au mépris de l’intégrité physique, à l’inconfort physique et aux douleurs, en plus de la possibilité de tirer des conclusions erronées en matière de violences sexuelles.

Les cliniciens chargés de procéder aux examens médico-légaux de victimes présumées d’agressions sexuelles, devraient être conscients du fait que la « normalité » de l’hymen ainsi que la valeur prédictive de l’examen de l’hymen, ne répondent à aucune définition standard et immuable. Les descriptions utilisant des termes comme « hymen intact » ou « hymen déchiré » devraient être systématiquement évitées. Les cliniciens devraient donner leurs conclusions en utilisant la terminologie et les normes internationales des caractéristiques morphologiques (voir schéma 1).

Vu que l’examen de l’hymen ne permet que rarement de déterminer si l’hymen ou le vagin a été pénétré par un pénis ou par un objet, sa valeur diagnostique ou médico-légale est faible voire nulle. Nous appelons les cliniciens à tenir compte de la très faible valeur prédictive de l’examen de l’hymen et : 1) éviter de s’en remettre uniquement aux caractéristiques de l’hymen dans les examens et les comptes rendus en matière d’agression sexuelle ; 2) faciliter la prise de conscience de ce problème chez leurs homologues et collègues des forces de l’ordre et du système judicaire ; et 3) encourager, au cours de la formation clinique de tous les spécialistes impliqués dans la santé sexuelle ou reproductive des femmes et des filles, les discussions factuelles sur les limites de l’examen de l’hymen.

Déclarations

Approbation déontologique et accord de collaboration : pas applicable

Accord pour la publication : pas applicable

Disponibilité des données pertinentes : pas applicable. Le partage des données ne s’applique pas à cet article car aucune donnée n’a été générée ou analysée au cours de cette étude.

Conflits d’intérêts : aucun

Divulgation : les docteurs Mishori, Ferdowsian, et Volpellier sont des consultants rémunérés du Programme sur les violences sexuelles en zones de conflit de Physicians for Human Rights.

Financement : le Programme sur les violences sexuelles en zones de conflit de Physicians for Human Rights remercie, pour leur soutien, le Fonds d’affectation spéciale des Nations Unies pour l’élimination de la violence à l’égard des femmes, The Sigrid Rausing Trust, NoVo Foundation, Pro Victimis Foundation et the Open Society Initiative for East Africa.

Contributions d’auteurs : idée (RM, HF, KN) ; recherche et synthèse documentaire (RM, HF, MV) ; écriture et révisions du texte (RM, HF, MV, KN, TM) ; tableaux et schémas (RM, TM) ;

Remerciements

Nous souhaitons remercier les personnes qui ont contribué à la création de ce texte ou qui ont vérifié les versions antérieures : Stefan Schmitt, Dallas Mazoori, Susannah Sirkin, Michele Curtis, Elizabeth Novick.

Sources

1. Schuster, S. (2015). Hymen Restoration: “My” Discomfort, “Their” Culture, and Women’s Missing Voice. The Journal of Clinical Ethics, 26(2), 162–165.
2. Eich, T. (2010). A tiny membrane defending “us” against “them”: Arabic Internet debate about hymenorraphy in Sunni Islamic law. Culture, Health & Sexuality, 12(7), 755–769.
3. Hobday, A. J., Haury, L., & Dayton, P. K. (1997). Function of the human hymen. *Medical Hypotheses*, *49*(2), 171–173.
4. What’s the Purpose of the Human Hymen? | DiscoverMagazine.com. (n.d.). Retrieved February 18, 2019, from <http://discovermagazine.com/2014/nov/3-ask-discover>
5. Independent Forensic Expert Group. (2015a). Statement on virginity testing. Journal of Forensic and Legal Medicine, 33, 121–124
6. Olson, RM., García-Moreno, C. (2017). Virginity testing: a systematic review. Reproductive Health, 14, 61.
7. World Health Organization. Interagency statement calls for the elimination of “virginity-testing”. October 18, 2018. Retrieved February 20, 2109. <https://www.who.int/reproductivehealth/virginity-testing-elimination/en/>
8. Isis Yazidi sex slaves subjected to traumatic “virginity tests” after escaping | The Independent. (n.d.). Retrieved July 5, 2017, from https://www.independent.co.uk/news/world/middle-east/isis-yazidi-sex-slaves-subjected-to-traumatic-virginity-tests-after-escaping-a6843446.html
9. Paradise, JE. “Predictive Accuracy and the Diagnosis of Sexual Abuse: A Big Issue about a Little Tissue.” *Child Abuse & Neglect* 1989. 13 (2): 169–76.
10. Berenson, AB., Chacko, MR., Wiemann, CM., Mishaw, CO., Friedrich, WN, Grady, JJ. (2002). Use of hymenal measurements in the diagnosis of previous penetration. Pediatrics, 109(2), 228–235.
11. Feeney, N. (2014, February 7). Living Myths About Virginity. Retrieved February 23, 2019, from <https://www.theatlantic.com/health/archive/2014/02/living-myths-about-virginity/283628/>
12. Heger, AH., Ticson, L., Guerra, L., Lister, J., Zaragoza, T., McConnell, G., Morahan, M. (2002). Appearance of the genitalia in girls selected for nonabuse: review of hymenal morphology and nonspecific findings. Journal of Pediatric and Adolescent Gynecology, 15(1), 27–35.
13. Imperforate Hymen: Background, Problem, Epidemiology. (2017). Retrieved from <https://emedicine.medscape.com/article/269050-overview>
14. Jacobs, AM, Alderman, EM. (2014). Gynecologic Examination of the Prepubertal Girl. Pediatrics in Review, 35(3), 97–105.
15. Berenson, A., Heger, A., Andrews, S. (1991). Appearance of the hymen in newborns. Pediatrics, 87(4), 458–465.
16. Mor, N., Merlob, P. (1988). Congenital absence of the hymen only a rumor? Pediatrics, 82(4), 679–680.
17. Kimberley, N., Hutson, JM., Southwell, BR., Grover, SR. (2012). Vaginal agenesis, the hymen, and associated anomalies. Journal of Pediatric and Adolescent Gynecology, 25(1), 54–58.
18. Berenson, AB. (1995). A longitudinal study of hymenal morphology in the first 3 years of life. Pediatrics, 95(4), 490–496.
19. Royal College of Paediatrics and Child Health: The Physical Signs of Child Sexual Abuse: an Evidence-Based Review and Guidance for Best Practice (2011)  Aug 2011).pdf. (n.d.). Retrieved from https://www.rcem.ac.uk/docs/External%20Guidance/10z9.%20The%20Physical%20Signs%20of%20Child%20Sexual%20Abuse%20-%20Interim%20Statement(RCPCH,%20Aug%202011).pdf.
20. Curtis, E., San Lazaro, C. (1999). Appearance of the hymen in adolescents is not well documented. BMJ (Clinical Research Ed.), 318(7183), 605.
21. Rogers, DJ., Stark, M. (1998). The hymen is not necessarily torn after sexual intercourse. BMJ (Clinical Research Ed.), 317(7155), 414.
22. Whitley, N. (1978). The first coital experience of one hundred women. JOGN Nursing; Journal of Obstetric, Gynecologic, and Neonatal Nursing, 7(4), 41–45.
23. Loeber O. “Over het zwaard en de schede; bloedverlies en pijn bij de eerste coïtus; Een onderzoek bij vrouwen uit diverse culturen” [About the Sword and the Sheet; Blood Loss and Pain at First Coitus. A study of Women with Different Cultural Backgrounds], Tijdschrift voor Seksuologie, vol. 32, (2008): 129
24. Hornor, G. (2010). A Normal Ano-genital Exam: Sexual Abuse or Not? Journal of Pediatric Health Care, 24(3), 145–151. https://doi.org/10.1016/j.pedhc.2008.10.007
25. McCann, J., Voris, J., Simon, M., Wells, R. (1989). Perianal findings in prepubertal children selected for nonabuse: a descriptive study. Child Abuse & Neglect, 13(2), 179–193.
26. A National Protocol for Sexual Abuse Medical Forensic Examinations Pediatric. April 2016. https://www.justice.gov/ovw/file/846856/download Accessed 7-4-17.
27. Kellogg, ND., Menard, SW., Santos, A. (2004a). Genital anatomy in pregnant adolescents: “normal” does not mean “nothing happened.” Pediatrics, 113(1 Pt 1), e67-69
28. Adams, JA., Botash, AS., & Kellogg, N. (2004). Differences in hymenal morphology between adolescent girls with and without a history of consensual sexual intercourse. Archives of Pediatrics & Adolescent Medicine, 158(3), 280–285. <https://doi.org/10.1001/archpedi.158.3.280>
29. Berkowitz, C. D. (2011). Healing of genital injuries. Journal of Child Sexual Abuse, 20(5), 537–547.
30. Goodyear-Smith, FA, Laidlaw, TM. (1998). What is an “intact” hymen? A critique of the literature. Medicine, Science, and the Law, 38(4), 289–300. https://doi.org/10.1177/002580249803800404
31. Goodyear-Smith, FA., Laidlaw, TM. (1998). Can tampon use cause hymen changes in girls who have not had sexual intercourse? A review of the literature. Forensic Science International, 94(1), 147–153. .
32. McCann, J., Wells, R., Simon, M., & Voris, J. (1990). Genital findings in prepubertal girls selected for nonabuse: a descriptive study. Pediatrics, 86(3), 428–439.
33. Volpellier, M. (2009). Physical forensic signs of sexual torture in children. A guideline for non specialized medical examiners. Torture: Quarterly Journal on Rehabilitation of Torture Victims and Prevention of Torture, 19(2), 157–166
34. McCann, J., Miyamoto, S., Boyle, C., & Rogers, K. (2007). Healing of hymenal injuries in prepubertal and adolescent girls: a descriptive study. Pediatrics, 119(5), e1094-1106.
35. M, Gall, John A.. (03/14/2011). The Paediatric Hymen. Current practice in forensic medicine. Wiley-Blackwell. 43 - 61-61. (ISBN: 0-470-74487-1, 978-0-470-74487-1). (n.d.).
36. Modelli, MES., Galvão, MF., Pratesi, R. (2012). Child sexual abuse. Forensic Science International, 217(1–3), 1–4.
37. Adams, JA., Girardin, B., Faugno, D. (2001). Adolescent Sexual Assault. Journal of Pediatric and Adolescent Gynecology, 14(4), 175–180.
38. Adams, Girardin, & Faugno, (2000). Signs of genital trauma in adolescent rape victims examined acutely. Journal of Pediatric and Adolescent Gynecology, 13(2), 88.
39. Adams, J. A. (2013). Signs of recent or healed injury to the genitalia in prepubertal girls describing penile-vaginal contact are uncommon. Medicine, Science, and the Law, 53(2), 117–118.
40. Biggs, M., Stermac, LE., & Divinsky, M. (1998). Genital injuries following sexual assault of women with and without prior sexual intercourse experience. CMAJ: Canadian Medical Association Journal, 159(1), 33–37.
41. The Royal College of Pediatrics and Child Health: The Physical Signs of Child Sexual Abuse: an evidence-based review and guidance for best practice (2015) p 54, p 57
42. Adams JD, Farst KJ, Kellogg ND,Interpretation of Medical Findings in Suspected Child Sexual Abuse: An Update for 2018. [J Pediatr Adolesc Gynecol.](https://www.ncbi.nlm.nih.gov/pubmed/29294380) 2018 Jun;31(3):225-231
43. A National Protocol for Sexual Assault medical Forensic Examinations. Adults and Pediatrics. 2013 & 2016. US Department of Justice. Washington, DC. Accessed 10-15-18. <https://www.justice.gov/ovw/file/846856/download> <https://www.ncjrs.gov/pdffiles1/ovw/241903.pdf>
44. Adams, J. (2003). Normal studies are essential for objective medical evaluations of children who may have been sexually abused. Acta Pædiatrica, 92(12), 1378–1380. https://doi.org/10.1111/j.1651-2227.2003.tb00818.x
45. Unintentional Perineal Injury in Prepubescent Girls: A Multicenter, Prospective Report of 56 Girls | Articles | Pediatrics. (n.d.). Retrieved July 5, 2017, from http://pediatrics.aappublications.org.proxy.library.georgetown.edu/content/95/5/628
46. World Health Organization, Health Care for Women Subjected to Intimate Partner Violence or Sexual Violence; World Health Organization, Guidelines for Medico-Legal Care for Victims of Sexual Violence. WHO/RHR/14.26 Geneva, Switzerland. <http://apps.who.int/iris/bitstream/10665/136101/1/WHO_RHR_14.26_eng.pdf?ua=1> Accessed 07-05-17
47. Jean-Jacques Amy, “Certificates of virginity and reconstruction of the hymen,” European Journal of Contraception and Reproductive Healthcare, vol. 13, no. 2, (Jun. 2008): 111–112).
48. Faikoglu, R., Yükseloglu, H., Ozcan, S., Petridis, G., Tari, I., & Kalfoglou, E. A. (2007). The gynaecologist as an expert witness in cases of sexual abuse. Reproductive Biomedicine Online, 15 Suppl 1, 41–42.
49. Sexual Abuse, Child | The Rational Clinical Examination: Evidence-Based Clinical Diagnosis | JAMAevidence | McGraw-Hill Medical. (n.d.). Retrieved February 20, 2019, from <https://jamaevidence.mhmedical.com/content.aspx?bookid=845&sectionid=61357599>
50. DiVasta, A. D., & Grace, E. (2013). Normal Hymen and Hymenal Variations. In *Practical Pediatric and Adolescent Gynecology* (pp. 43–48). John Wiley & Sons, Ltd. <https://doi.org/10.1002/9781118538555.ch9>
51. Brown, VW., Lamb, SM., Perkins, AM., Naim, DW. Starling, SP. (2014a). Knowledge regarding hymens and the sex education of parents. Journal of Child Sexual Abuse, 23(6), 674–689.
52. Faikoglu, R., Yükseloglu, H., Ozcan, S., Petridis, G., Tari, I., & Kalfoglou, E. A. (2007). The gynaecologist as an expert witness in cases of sexual abuse. Reproductive Biomedicine Online, 15 Suppl 1, 41–42.
53. Dabson, AM., Magin, PJ., Heading, G., Pond, D. (2014). Medical students’ experiences learning intimate physical examination skills: a qualitative study. BMC Medical Education, 14, 39.
54. McBain, L., Pullon, S., Garrett, S., & Hoare, K. (2016). Genital examination training: assessing the effectiveness of an integrated female and male teaching programme. *BMC Medical Education*, *16*(1), 299. <https://doi.org/10.1186/s12909-016-0822-y>
55. Powell, H. S., Bridge, J., Eskesen, S., Estrada, F., & Laya, M. (2006a). Medical students’ self-reported experiences performing pelvic, breast, and male genital examinations and the influence of student gender and physician supervision. *Academic Medicine: Journal of the Association of American Medical Colleges*, *81*(3), 286–289.
56. Bhoopatkar, H., Wearn, A., & Vnuk, A. (2017). Medical students’ experience of performing female pelvic examinations: Opportunities and barriers. *The Australian & New Zealand Journal of Obstetrics & Gynaecology*, *57*(5), 514–519. <https://doi.org/10.1111/ajo.12634>
57. Association of Professors of Gynecology and Obstetrics (APGO) Clinical Skills Curriculum - The Pelvic Exam - https://www.apgo.org/educational-resources/basic-clinical-skills/pelvic-exam-2017/ Accessed 7-5-17
58. Edelman, A., Anderson, J., Lai, S., Braner, D. A. V., & Tegtmeyer, K. (2007). Pelvic Examination. New England Journal of Medicine, 356(26),
59. Abdulghani, H. M., Haque, S., Irshad, M., Al-Zahrani, N., Al-Bedaie, E., Al-Fahad, L., … Al-Mohaimeed, A. (2016). Students’ perception and experience of intimate area examination and vie sexuelle taking during undergraduate clinical skills training: A study from two Saudi medical colleges. *Medicine*, *95*(30), e4400. <https://doi.org/10.1097/MD.0000000000004400>
60. Alnassar, S. A., Almuhaya, R. A., Al-Shaikh, G. K., Alsaadi, M. M., Azer, S. A., & Isnani, A. C. (2012). Experience and attitude of interns to pelvic and sensitive area examinations during their undergraduate medical course. *Saudi Medical Journal*, *33*(5), 551–556
61. Bates, C. K., Carroll, N., & Potter, J. (2011). The Challenging Pelvic Examination. Journal of General Internal Medicine, 26(6), 651–657
62. Braverman, PK., Breech, L., & Committee on Adolescence. (2010). American Academy of Pediatrics. Clinical report--gynecologic examination for adolescents in the pediatric office setting. Pediatrics, 126(3), 583–590
63. Loveless, M. B., Finkenzeller, D., Ibrahim, S., & Satin, A. J. (2011). A simulation program for teaching obstetrics and gynecology residents the pediatric gynecology examination and procedures. Journal of Pediatric and Adolescent Gynecology, 24(3), 127–136
64. Dumont, T., Hakim, J., Black, A., & Fleming, N. (2015). Does an Advanced Pelvic Simulation Curriculum Improve Resident Performance on a Pediatric and Adolescent Gynecology Focused Objective Structured Clinical Examination? A Cohort Study. Journal of Pediatric and Adolescent Gynecology
